# Draft Genome Sequence of the Field Isolate *Brucella melitensis* Strain Bm IND1 from India

**DOI:** 10.1128/genomeA.00497-14

**Published:** 2014-05-29

**Authors:** Sashi Bhushan Rao, Vivek K. Gupta, Mukesh Kumar, Nagendra R. Hegde, Gary A. Splitter, Pallu Reddanna, Girish K. Radhakrishnan

**Affiliations:** a Ella Foundation, Genome Valley, Hyderabad, India; b Central Institute for Research on Goats, Makhdoom, Mathura, India; c Indian Agricultural Research Institute, Pusa Campus, New Delhi, India; d University of Wisconsin-Madison, Madison, Wisconsin, USA; e National Institute of Animal Biotechnology, Hyderabad, India

## Abstract

*Brucella* spp. are facultative intracellular bacterial pathogens causing the zoonotic disease brucellosis. Here, we report the draft genome sequence of the *Brucella melitensis* strain from India designated Bm IND1, isolated from stomach contents of an aborted goat fetus.

## GENOME ANNOUNCEMENT

Brucellosis is an economically important livestock disease and a major reemerging zoonosis that is difficult to diagnose and treat. Globally, about 500,000 new human brucellosis cases are reported every year, causing substantial residual disability and travel-associated morbidity (1, 2). No vaccine is currently available for human use to treat this disease, and the available animal vaccines are infectious to humans and have many side effects. The significance of the disease is its zoonotic impact on people and the economic loss caused by livestock disease and reproductive failures. There are 10 species of *Brucella* based on their host tropism: *B. melitensis* (sheep and goats), *B. abortus* (cattle), *B. suis* (swine), *B. canis* (dogs), *B. ovis* (sheep), *B. neotomae* (wood rats), *B. ceti* (dolphins, porpoises, and whales), *B. pinnipedialis* (seals), *B. microti* (voles and foxes), and *B. inopinata* (humans). The first four species are pathogenic to humans in decreasing order of severity.

The pathogenicity of *Brucella* spp. is due to the ability to adapt to environmental conditions encountered intracellularly, though they lack classical virulence factors (3, 4). The mechanisms that mediate the invasion and intracellular persistence of *Brucella* are poorly characterized. Therefore, gaining an improved understanding of the virulence factors may enable the development of novel therapeutic and preventive strategies for brucellosis. Whole-genome sequencing and comparative genome analysis are important first steps in this direction.

Brucellosis is endemic in India. Previous studies have indicated a high seroprevalence of human brucellosis from various parts of India, including Andhra Pradesh (11.51%) and Punjab (26.6%) (5). However, little information is available regarding genetic variation and epidemiology, other than the recent publication of the genome of *B. melitensis* strain ADMAS-G1 (6).

We isolated a *B. melitensis* strain from the stomach contents of an aborted goat fetus and designated it Bm IND1. Preliminary analysis of the 16S rRNA sequence of this strain revealed 100% similarity with the genus *Brucella*, and further analyses by PCR for *omp31* confirmed it to belong to *B. melitensis* (7, 8). The genomic DNA of Bm IND1 was isolated, and whole-genome shotgun sequencing was performed using the Illumina HiSeq platform to achieve 100× coverage. A total of 102 contigs with a sequence length compliance of *N*_50_ at 6.3 kb were assembled using the SSAKE software, and gene prediction was done by GeneMark.hmm (9, 10). The draft whole-genome sequence of the strain Bm IND1 was found to have 3,284,360 bases, 3,360 protein-coding genes, and a G+C content of 57.2%. Further, gene functions were annotated by the KEGG, Pfam, and Clusters of Orthologous Groups (COG) databases. Secretory peptides were identified with SignalP (11–14).

The Bm IND1 genome harbors 49 tRNAs, 3 rRNAs, and 964 KEGG orthologs. There were 1,724 and 1,022 genes identified in the Pfam and COG databases, respectively. In addition, 58 genes were found to be secretory in nature and might be involved in host-pathogen interactions. Further analysis and experimental confirmation will be described in future studies.

### Nucleotide sequence accession numbers.

This whole-genome shotgun project has been deposited at DDBJ/EMBL/GenBank under the accession no. JMKL00000000. The version described in this paper is version JMKL01000000.
